# In Situ and Real-Time Nanoscale Monitoring of Ultra-Thin Metal Film Growth Using Optical and Electrical Diagnostic Tools

**DOI:** 10.3390/nano10112225

**Published:** 2020-11-09

**Authors:** Jonathan Colin, Andreas Jamnig, Clarisse Furgeaud, Anny Michel, Nikolaos Pliatsikas, Kostas Sarakinos, Gregory Abadias

**Affiliations:** 1 Institut Pprime, UPR 3346, CNRS-Université de Poitiers-ENSMA, 11 Boulevard Marie et Pierre Curie, TSA 41123, CEDEX 9, 86073 Poitiers, France; JJCOLIN@protonmail.com (J.C.); andreas.jamnig@liu.se (A.J.); cfurgeaud@posta.unizar.es (C.F.); anny.s.michel@univ-poitiers.fr (A.M.); 2 Nanoscale Engineering Division, Department of Physics, Chemistry and Biology, Linköping University, SE 581 83 Linköping, Sweden; nikolaos.pliatsikas@liu.se

**Keywords:** real-time monitoring, polycrystalline film growth, growth dynamics, interface reactivity, adatom mobility, wafer curvature, electrical resistance, spectroscopic ellipsometry

## Abstract

Continued downscaling of functional layers for key enabling devices has prompted the development of characterization tools to probe and dynamically control thin film formation stages and ensure the desired film morphology and functionalities in terms of, e.g., layer surface smoothness or electrical properties. In this work, we review the combined use of in situ and real-time optical (wafer curvature, spectroscopic ellipsometry) and electrical probes for gaining insights into the early growth stages of magnetron-sputter-deposited films. Data are reported for a large variety of metals characterized by different atomic mobilities and interface reactivities. For fcc noble-metal films (Ag, Cu, Pd) exhibiting a pronounced three-dimensional growth on weakly-interacting substrates (SiO_2_, amorphous carbon (a-C)), wafer curvature, spectroscopic ellipsometry, and resistivity techniques are shown to be complementary in studying the morphological evolution of discontinuous layers, and determining the percolation threshold and the onset of continuous film formation. The influence of growth kinetics (in terms of intrinsic atomic mobility, substrate temperature, deposition rate, deposition flux temporal profile) and the effect of deposited energy (through changes in working pressure or bias voltage) on the various morphological transition thicknesses is critically examined. For bcc transition metals, like Fe and Mo deposited on a-Si, in situ and real-time growth monitoring data exhibit transient features at a critical layer thickness of ~2 nm, which is a fingerprint of an interface-mediated crystalline-to-amorphous phase transition, while such behavior is not observed for Ta films that crystallize into their metastable tetragonal β-Ta allotropic phase. The potential of optical and electrical diagnostic tools is also explored to reveal complex interfacial reactions and their effect on growth of Pd films on a-Si or a-Ge interlayers. For all case studies presented in the article, in situ data are complemented with and benchmarked against ex situ structural and morphological analyses.

## 1. Introduction

Metal films with thicknesses of ~10 nm and below are ubiquitous in many modern life technologies, including microelectronics, displays, sensors, and energy storage/saving/conversion devices [1,2,3,4,5,6,7,8]. Such ultra-thin layers may form a continuous structure and fully wet underlying substrates or self-assemble into discrete nanoscale particles forming a discontinuous morphology. The latter film morphology (i.e., supported nanoparticles) is relevant for the field of heterogeneous catalysis and plasmonics, whereby nanoparticles with high surface-to-volume ratios and unique optical response emanating from localized surface plasmon resonance (LSPR) are leveraged in a broad range of applications [1,9,10,11,12,13,14,15]. Concurrently, fabrication of continuous and ultrasmooth metallic layers at thicknesses below 10 nm is desirable for opto-electronic applications which rely on, e.g., conductive transparent electrodes [6,16,17,18].

A significant fraction of thin films is today synthesized via vapor condensation, a robust and versatile method routinely employed in industry and research laboratories. Material from a solid or liquid source is vaporized using physical and/or chemical means (e.g., by heating or momentum transfer); vapor is transported through the gas phase and condenses on a substrate where it forms a film. During the condensation process, the flux of atoms (and molecules) from the vapor to the solid substrate surface is typically multiple orders of magnitude larger than the flux of material returning from the substrate surface to the vapor phase. This flux difference (also known as supersaturation at the vapor/solid interface) leads to excess of atoms on the substrate, so that film-forming species do not have sufficient time to self-assemble into minimum-energy configurations predicted by thermodynamics. It is then said that film formation proceeds far from thermodynamic equilibrium and the resulting film morphology and microstructure are determined by the occurrence rates (i.e., kinetics) of atomic-scale structure-forming mechanisms [19,20,21,22,23,24,25]. Further aspects, crucial for film growth, are chemical reactions (i.e., compound formation) and intermixing at the film/substrate interface, which depend not only on kinetics but are also governed by the thermodynamics (i.e., miscibility vs. immiscibility) of the materials involved [26,27,28,29].

Film physical attributes are closely linked with mesoscale morphological and nanoscale structural features, including grain/island size and shape, crystal structure and orientation, and surface roughness. Such features are difficult to predict a priori because they are determined by a complex interplay among a multitude of deposition process parameters, as well as by film/substrate interactions. Hence, the use of robust and non-destructive characterization tools that can provide information at the nanoscale is required for establishing the correlation among atomic-scale mechanisms and resulting film morphology. Implementing such techniques during synthesis (i.e., in situ and in real-time) is particularly advantageous, since it allows one to selectively study dynamic growth processes and decouple them from post-growth microstructural changes.

A wide palette of techniques for in situ thin-film growth monitoring is nowadays available and can be grouped into different categories, based on the measured physical quantities and operation principle (real-space imaging, diffraction, spectroscopy). Scanning probe microscopy (SPM) techniques provide a direct observation of atoms and clusters, as well as of their mobility, through real-space imaging of the film surface electronic density with sub-Ångström vertical resolution. As such, valuable information on atomic-scale mechanisms and their rates (e.g., diffusion barriers) can be obtained. However, SPM techniques are inherently restricted to the characterization of the island nucleation and growth stages at sub-monolayer metal coverage [4,30], require an ultra-high vacuum environment, and data acquisition rate is seldom compatible with a real-time growth monitoring [31,32].

Methods relying on low-energy electron microscopy (LEEM) provide access to mesoscopic lateral length scales (2–150 µm), with video-rate imaging amenable to studying dynamic processes on surfaces, but their lowest lateral resolution is of the order of 10 nm [33,34,35]. Crystal structure and film growth mode can be studied using reciprocal-space electron-based diffraction techniques, such as low-energy electron diffraction (LEED) or reflection high-energy electron diffraction (RHEED). LEEM, RHEED and LEED are surface-sensitive probes that they are ideally suited to study growth up to few monolayers (ML). They require ultra-high vacuum conditions and, hence, they are usually implemented to investigate thin film growth by thermal evaporation, although experimental setups based on differential pumping stages can be designed to be compatible with sputter-deposition. The aforementioned SPM and electron scattering techniques are often not directly integrated into deposition chambers; hence analysis is performed in a “stop-and-growth” fashion, in which a certain amount of metal deposit is iteratively probed at key film formation stages in separate analysis chambers.

Non-invasive, surface sensitive techniques based on X-rays can be advantageously employed to study the structural, morphological and chemical evolution during thin film growth [36,37,38,39,40,41,42,43]. X-ray reflectivity (XRR), grazing incidence small-angle X-ray scattering (GISAXS), X-ray diffraction (XRD), X-ray fluorescence (XRF), and X-ray absorption spectroscopy (XAS) can be used remotely to probe the sample surface, with the only requirement being that the deposition chamber must be equipped with X-ray transparent windows (such as beryllium or Kapton). These methods can be used separately or coupled to each another, but most in situ experiments during film deposition require synchrotron-based X-rays. The high-brilliance of third-generation synchrotron sources, along with modern fast two-dimensional (2D) X-ray detectors, facilitate the monitoring of the kinetics of thin film growth in real-time with fast-acquisition (milliseconds) and sub-monolayer precision. Besides, the ability to tune the photon energy to a specific experiment and material system is an additional asset.

Another category of in situ and real-time diagnostics is based on measuring the change of electrical and optical properties of the deposited layer as a function of time. Evolution of electrical properties (e.g., film resistivity) can be measured using four-point probe techniques [44,45,46,47], while typical optical diagnostics include reflectance spectroscopy [48,49,50,51] and spectroscopic ellipsometry [52,53,54,55,56,57,58,59,60]. These techniques can characterize all relevant film-growth stages up to the formation of a continuous layer and beyond, while they provide morphological information over mesoscopic length scales. They are also characterized by conceptual and practical simplicity, they are readily available in and compatible with typical thin-film synthesis apparatuses, and data interpretation is in most cases straightforward.

In the present review article, we demonstrate the strength of combining laboratory-scale electrical and optical in situ and real-time diagnostic tools for shedding light onto morphological evolution, structure formation, and growth dynamics in a wide gamut of film/substrate systems, whereby films are grown by physical vapor deposition techniques. In a first group of film/substrate systems, we study Ag, Cu, and Pd growth (all exhibiting fcc crystal structure) on a number of substrates, including Si covered with its native oxide layer, Si covered with a thermally grown SiO_2_ layer, and amorphous carbon (a-C). These film/substrate combinations exhibit minimum chemical interactions and reactivity, which allows us to selectively study the effect of atomic-scale kinetics on film growth. An additional effect of the weak film/substate interaction in the latter systems is that the deposited layers grow in a pronounced three-dimensional (3D) fashion, which offers an ideal test bed for identifying subtle changes of film morphology as a function of deposition conditions and material characteristics using optical and electrical probes [1,61,62]. As such, kinetics is studied both in terms of *intrinsic* atomic mobility of the thin-film materials—as approximated by their melting point $T_{m}$, which yields homologous temperatures $T_{h}=T/T_{m}$ of 0.24 (Ag), 0.22 (Cu), 0.16 (Pd and Fe), 0.1 (Mo) and 0.09 (Ta), at T = 300 K, where T is the substrate temperature [21,63,64,65]—and *extrinsic* deposition parameters, including deposition temperature and rate. In a second group, the importance of interface reactivity on film structure formation is addressed by discussing the growth of bcc transition metals (Fe, Mo and Ta) on amorphous Si (a-Si). Interfacial reactions are further examined by monitoring the growth of Pd films on a-Si and a-Ge layers. The selected metal/Si systems span a wide range of chemical reactivities. Although interface reaction and silicide formation during thermal annealing is well documented in the literature [26,66], studies on the nucleation processes during metal deposition on a-Si are scarce.

The content of the article is predominantly based on results generated by us over the past years and focused on metal films synthesized by magnetron sputtering using Ar plasma discharges. Our results are critically complemented by literature data, in order to expand the scope and relevance of our conclusions.

The article is organized as follows: Section 2 explains the overall strategy for thin-film synthesis and provides a brief description of the techniques used for in situ and real-time growth monitoring; Section 3 demonstrates the use of in situ an real-time techniques for studying morphological evolution and growth dynamics of metals on weakly-interacting substrates; Section 4 addresses the effect of interfacial reactivity on film morphological evolution as established by in situ and real-time methodologies; Section 5 summarizes the article and presents an outlook for future developments in the field.

## 2. Film Synthesis and Real-Time Growth Monitoring

### 2.1. Film Synthesis and In Situ/Real-Time Monitoring Strategy

**Figure 1:**
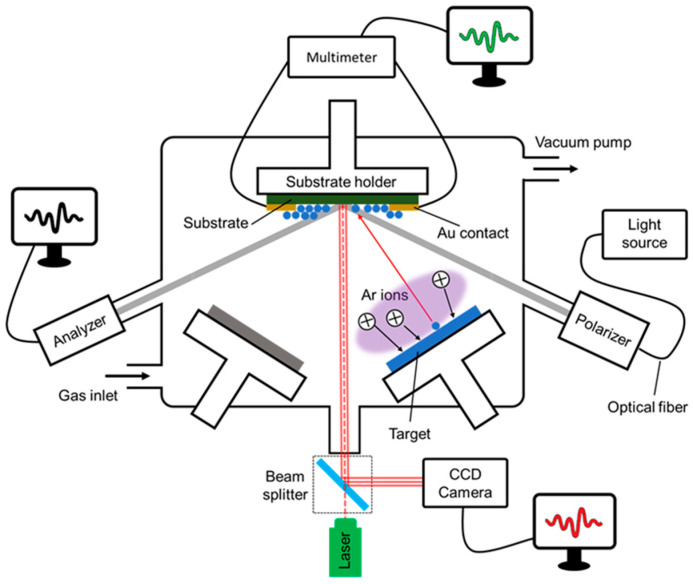
Generic schematic illustration of the sputter-deposition chamber used for collecting data reported in this work. The chamber is equipped with several in situ diagnostics which allow real-time growth monitoring: the wafer curvature set-up is attached at the bottom flange of the chamber and consists of a multiple-beam laser illuminating the substrate at near-normal incidence; the set-up for spectroscopic ellipsometry, operating at an incidence angle of ~70°, consists of a light source, polarizer, and analyzer. The sample holder stage can be fitted with a custom-built four-point probe apparatus to measure the change in electrical resistance during deposition.

Thin-film growth was performed by means of magnetron sputtering in three vacuum chambers located at the University of Poitiers (France) and Linköping University (Sweden), all equipped with a load-lock sample transfer system and multiple cathodes arranged in a confocal configuration. Moreover, the vacuum chambers are specifically designed to host techniques for real-time monitoring of the deposition process. One of the film deposition setups (at the University of Poitiers) achieves high-vacuum conditions (base pressure ≤ 8 × 10^−6^ Pa using a cryogenic pump); it enables measurements of stress evolution during film growth using the wafer curvature method (details are given in Section 2.2), while it also allows for monitoring the change in the film electrical resistance via the four-point probe technique (details are given in Section 2.3) using a custom-built sample holder stage. We note that, due to geometrical constraints and specificity of sample dimensions, the two diagnostics cannot be used simultaneously. The other two deposition chambers (at Linköping University) achieve ultra-high-vacuum conditions (base pressures ~10^−7^–10^−8^ Pa using turbomolecular pumps) and feature transparent viewports for mounting a spectroscopic ellipsometer to monitor the change in optical response of the growing layer (see Section 2.4 for details). All vacuum systems are equipped with a resistive substrate heater, such that deposition temperature can be varied and temperature effects on film-forming processes can be investigated. Deposition flux is controlled by changing the electrical power applied to the magnetrons. The generic layout of the deposition apparatuses, along with the in situ diagnostic tools, is schematically depicted in Figure 1.

### 2.2. Wafer Curvature Method

The wafer curvature method is based upon measuring the variation of the substrate curvature Δκ induced by the existence of stress in the film that is attached to it. There are different ways to detect the change in curvature, but the most sensitive and easy to implement in situ during deposition is the method relying on the optical measurement of Δκ using laser deflectometry [67,68]. In this work, we report data that were recorded with a multiple-beam optical stress sensor (MOSS) set-up, designed by k Space Associates (Dexter, MI, USA) [69,70]. The main advantage of illuminating the sample with multiple beams simultaneously is to alleviate the sensitivity to ambient vibrations during data acquisition: when using a beam array, Δκ is calculated by measuring the relative spacings $\Delta d=d-d_{0}$ between adjacent spots, instead of recording the absolute position of one reflected beam. A 3 × 3 array of parallel beams, with initial spacing $d_{0}$, is created using two etalons (beam splitters), and the beams reflected off the substrate are detected on a CCD camera located at a distance *L* from the substrate. A dedicated software allows for accurate measurement of the variation in spot spacing d(t) as a function of time *t* with typical acquisition rate of 10 Hz. The change in curvature Δκ is then obtained from the expression

(1) $$\Delta \kappa \left(t\right)=\frac{\cos\alpha }{2L}\frac{\Delta d}{d_{0}}=\frac{\cos\alpha }{2L}\left(\frac{d\left(t\right)-d_{0}}{d_{0}}\right)$$

where α is the incidence angle of the laser beam with respect to the substrate normal. In the curvature measurement setup used in the present work, the laser illuminates the substrate at near-normal incidence (α~0°) and *L*~70 cm. It is noted that the accurate determination of the optical distance *L* is realized using a set of mirrors with known curvature.

The biaxial stress in the growing film at a distance (i.e., height) z from the film/substrate interface, σ(z), is directly obtained from Δκ using Stoney’s equation according to [70]

(2) $$\langle \sigma_{f}\rangle \times h_{f}=\int_{0}^{h_{f}}\sigma \left(z\right)dz=\frac{M_{s}h_{s}^{2}}{6}\Delta \kappa$$

where $\langle \sigma_{f}\rangle \times h_{f}$ is the stress-thickness product (also referred to as the force per unit width, expressed in N/m), $\langle \sigma_{f}\rangle$ is the average stress in the film at thickness $h_{f}$, and $h_{s}$ and $M_{s}$ are the thickness and biaxial modulus of the substrate, respectively. For the MOSS measurements presented herein, 100 ± 2 µm thick Si (001) substrates (with dimensions of 1 × 1 cm^2^) were used. The substrates were mounted loosely on a sample holder, such that free bending during growth is possible.

In the present work, the substrate curvature method is not merely used as a stress evaluation technique but also as a sub-nanometer-scale sensitive tool for real-time monitoring of film/substrate interfacial reactions, island nucleation, island coalescence, and overall film morphological evolution [71,72]. For instance, thin films growing in a 3D fashion exhibit a characteristic compressive-tensile-compressive stress evolution with increasing film thickness, and the position of the tensile peak maximum has been shown to coincide with the thickness $h_{cont}$ at which a continuous layer is formed [73].

### 2.3. Electrical Resistance

The second in situ diagnostic that is implemented to monitor film growth evolution is a custom-built apparatus with four-point probe (4PP) arrangement for measuring the variation of the film sheet resistance $R_{s}$ during deposition. The setup consists of a sample-holder stage, compatible with transfer system from the load-lock to the main deposition chamber, and an electrical collector mounted in the main chamber. The collector is equipped with feedthrough connectors to a Keithley sourcemeter that is interfaced to a PC and controlled by a dedicated software. This setup allows measurements on a series of samples without venting the main chamber. The stainless-steel substrate holder is insulated from the substrate using a 6 mm thick Teflon disk, which can be heated from the back side using a resistive heater. Gold contacts are pre-deposited on the Si substrate, and a conical mask is used during metal film growth to protect the contacts from the vapor flux. More details on the in situ resistivity set-up can be found in [47]. Growth is performed on 350 µm thick, highly-resistive (with resistivity in the range 1–5 kΩ·cm) Si wafers to maximize the change in electrical resistance upon metallic film deposition. We report here the evolution of $R_{s}\times h_{f}$ vs. deposition time (or film thickness $h_{f}$). Note that the quantity $R_{s}\times h_{f}$ is proportional to the film resistivity ρ, which is derived by applying a correction factor to account for the specific sample geometry. In this work, since the sample geometry remains unchanged, we will only report the raw data in the form of $R_{s}\times h_{f}$ vs. $h_{f}$ curves, from which two morphological transition thicknesses, i.e., the percolation ($h_{perc}$) and the continuous formation ($h_{cont}$) thicknesses are extracted.

### 2.4. Spectroscopic Ellipsometry

**Figure 2:**
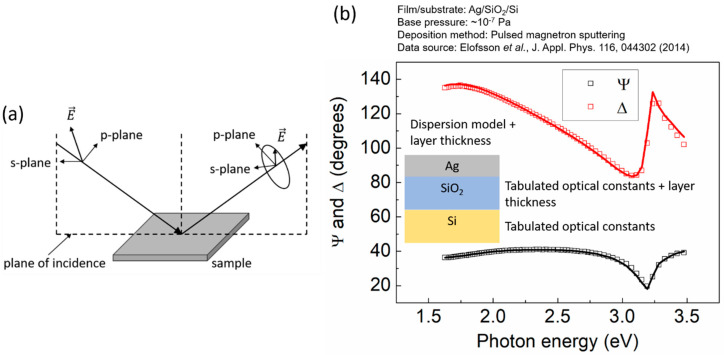
(**a**) Schematic illustration of the principle of spectroscopic ellipsometry. Linearly polarized light (with electric field vector $\vec{E}$) is reflected at sample surface. Reflection of the incident light causes change for the polarization state to elliptical. p-plane and s-plane indicate the planes that are parallel and perpendicular to the plane of incidence, respectively. By measuring the reflected intensity (i.e., intensity of the electric field $\vec{E}$ ) along the s and p directions, the ellipsometric angles Ψ and ∆ (amplitude ratio and phase shift, respectively, of the reflected light relative to the incident light) are determined from which the optical properties of the sample under investigation can be extracted. (**b**) Ψ and ∆ experimental data (square symbols) measured from an electrically conductive Ag film grown on Si substrate covered by thermally grown ~300 nm SiO_2_ layer (data are taken from [62]). The ellipsometric data depend in a complex manner on the optical properties of Si, SiO_2_, and Ag, as well as on the SiO_2_ and Ag layer thicknesses. Data are fitted using three-phase model (model data are represented by solid lines) which is schematically depicted in the inset with additional details provided in the text.

Spectroscopic ellipsometry (SE) is a non-destructive optical technique in which linearly or circularly polarized light is used to irradiate the sample under investigation [74]. Upon interaction (i.e., reflection or transmission) with the sample, the polarization state of light becomes elliptical. By measuring the change in the light polarization state, the optical properties of the sample can be determined. Figure 2a depicts schematically the concept of ellipsometry for the cases of linearly polarized incident light and reflection geometry. To describe the change in polarization, the reflectance is analyzed into the orthogonal s-p system, where s and p denote planes that are parallel and perpendicular to the plane of incidence, respectively. By measuring the reflected intensity (i.e., the intensity of the electric field $\vec{E}$) along the s and p directions, the ellipsometric angles Ψ and ∆ (amplitude ratio and phase shift, respectively, of the reflected light relative to the incident light) are determined. In the case of a bulk sample (i.e., a sample in which the incident light is only absorbed from and reflected at the sample/ambient interface), the complex dielectric function $\tilde{\varepsilon }\left(\omega \right)$ of the material under investigation can be computed directly from the quantities Ψ and ∆. The latter is not possible when the sample consists of a partially transparent film residing on the substrate, as in that case Ψ and ∆ depend in a non-trivial fashion on the optical response of the substrate and the film, as well as on the film thickness. Hence, the use of models is required for determining the optical properties of the thin film, as shown schematically in Figure 2b and explained hereafter.

In situ and real-time SE is used to monitor the evolution of the angles Ψ and ∆ over multiple wavelengths during film growth and, by using appropriate models, determine the changes of the optical properties of the deposited layer. These changes are then correlated with the overall film morphological evolution, as explained in detail in Section 3.

The model system that is used in the present article for demonstrating the ability of SE to study film growth is Ag/SiO_2_/Si. For such films, ellipsometric angles are acquired every ~2 s at 67 incident-light photon energies in the range 1.6–3.2 eV, at an angle of incidence of ~70° from the substrate normal (see representative curves in Figure 2b from [62]). The acquired data are fitted to a three-phase model consisting of substrate, film, and ambient (see Figure 2b). The substrate is modeled as a 625 µm-thick Si slab with a SiO_2_ overlayer, the thickness of which (in the range ~300 to 500 nm) is confirmed by measuring the optical response of the substrate prior to deposition. Reference data for the substrate layers are taken from Herzinger et al. [75]. The optical response of the film is described by the following dispersion models [76] depending on the film growth stage.

Discontinuous layer: During initial growth stages, the Ag films on SiO_2_ surface primarily self-assemble in discrete islands that support LSPR. Being a resonant effect, LSPR can be described by adapting the Lorentz oscillator model [55,77] to express the complex dielectric function of the layer $\tilde{\varepsilon }\left(\omega \right)$ as

(3) $$\tilde{\epsilon }\left(\omega \right)=\frac{f\omega_{0}{}^{2}}{\omega_{0}^{2}-\omega^{2}-i\Gamma \omega }$$

In Equation (3), f and $\omega_{0}$ are the oscillator strength and resonance frequency, respectively, and Γ represents the damping rate of the plasmon resonance. The position of LSPR $\omega_{0}$ is used to gauge changes of film morphology, including changes in substrate area coverage and island size (see Section 3.4).

Electrically conducting layer: The optical response of electrically conductive Ag films is described by the Drude free electron theory, which is extensively used for ideal metals [76]. In this case $\tilde{\varepsilon }\left(\omega \right)$ is given by the expression,

(4) $$\tilde{\varepsilon }\left(\omega \right)=\epsilon_{\infty }-\frac{\omega_{p}^{2}}{\omega^{2}+i\Gamma_{D}\omega }$$

In Equation (4), $\epsilon_{\infty }$ is a constant that accounts for the effect of interband transitions occurring at frequencies higher than the ones considered here, $\Gamma_{D}$ is the free-electron damping constant, and $\omega_{p}=\sqrt{ne^{2}/\varepsilon_{0}m_{e}}$ is the free-electron plasma frequency, where n is the free-electron density, $\varepsilon_{0}$ is the permittivity of free space, and $m_{e}$ is the free-electron effective mass. From Equation (4), the room-temperature film resistivity is calculated as

(5) $$\rho =\frac{\Gamma_{D}}{\epsilon_{0}\omega_{p}^{2}}$$

The evolution of resistivity as function of film thickness (the latter is also determined from SE) provides information with regards to continuous layer formation, the degree of 3D clustering and the dynamics of film growth.

## 3. Growth of Metal Films on Weakly-Interacting Substrates

### 3.1. Film Growth Stages and Morphological Transitions

**Figure 3:**
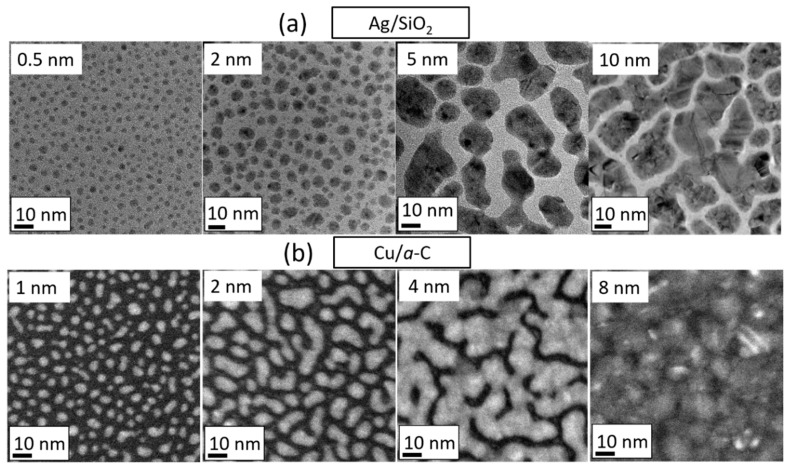
Plan-view TEM micrographs showing the morphological evolution and various formation stages of (**a**) Ag and (**b**) Cu thin films with different thickness deposited on SiO_2_ (Ag) and a-C (Cu) by magnetron sputtering at room temperature. Island nucleation and growth at 0.5 nm in (**a**); complete coalescence at 2 nm in (**a**); incomplete coalescence and formation of elongated islands at 5 and 10 nm in (**a**) and 1 and 2 nm in (**b**); hole-filling at 4 nm in (**b**); continuous-layer formation at 8 nm in (**b**). Micrographs in (**a**) correspond to bright-field images (Reprinted with permission from [79]. Copyright ACS 2020), while the images in (**b**) were taken using scanning transmission electron microscopy in high-angle annular dark field (STEM-HAADF) mode (Reprinted with permission from [78]).

The present section provides a brief description of formation stages and morphological evolution during polycrystalline film growth, with emphasis on weakly-interacting film/substrate systems. Growth starts with adsorption of vapor atoms (referred to as adatoms) on the substrate surface and formation of spatially separated single-crystalline islands via agglomeration of adatoms (nucleation), which grow in size (island growth) and impinge on each other forming new larger islands (coalescence). The process of coalescence also leads to a reduction of the island number density on the substrate surface and continues until the boundaries between single-crystalline islands (i.e., grains) become immobile, such that coalescence stops and a network of interconnected polycrystalline islands forms. Subsequent deposition fills the inter-island space with material (hole filling) and, once this process is completed, a continuous film is formed. The afore-mentioned stages can be visualized in Figure 3 which displays the sequence of transmission electron micrographs taken at various nominal thickness during sputter-deposition of Ag and Cu films on SiO_2_ and a-C substrates, respectively [78,79]. We note here that the nominal thickness $h_{f}$ corresponds to the amount of vapor deposited on the substrate surface at any given time t (irrespective of whether the film is discontinuous or continuous), and it is calculated as $h_{f}=F\times t$ with F being the deposition rate as determined by the thickness of a continuous layer (e.g., from XRR).

Throughout the various film formation stages, competing atomic-scale processes are operative, giving rise to characteristic morphological transitions, which provide information on the degree of 3D clustering (which is inherent in weakly-interacting film/substrate systems) and the overall growth dynamics. These transitions are explained in the following.

Island density saturation: At finite temperatures, adatoms perform a two-dimensional random walk on the substrate surface with a diffusivity D, a quantity that depends on the potential energy landscape encountered by the adatoms and on the growth temperature. Vapor deposition increases the adatom number density on the substrate surface until adatom-adatom encounters lead to nucleation, i.e., the formation of stable atomic clusters (islands) [22]. Nucleation results in an increase of the island number density N on the substrate, until a saturation value $N_{sat}$ is reached. The magnitude of $N_{sat}$ is governed by the competition among formation of new islands and incorporation of adatoms to existing ones, and it is expressed as

(6) $$N_{sat}\propto \;{\left(\frac{F}{D}\right)}^{x}$$

where $x=\frac{1}{3}\left(\frac{2}{7}\right)$ for 2D (3D) islands [22,80,81]. Increase of (e.g., caused by increasing the deposition temperature *T*) leads to larger adatom mean free path on the substrate surface. This favors adatom incorporation into existing islands, at the expense of nucleating new ones, and it results in a decrease of $N_{sat}$ Conversely, increase of F leads to a larger adatom number density on the substrate surface. This increases the probability of adatom–adatom encounters and, hence, promotes island nucleation at the expense of island growth, resulting in a larger $N_{sat}$.

Elongation transition: Islands grow larger by incorporation of adatoms and/or material from the vapor phase. Beyond $N_{sat}$, island growth becomes the main process that determines film morphology by increasing the fraction of substrate surface covered by the deposit. This is until two or more islands impinge and coalesce into a larger single-crystalline island, which largely erases morphological features attained during earlier stages of film growth. The time required for the coalescing islands to re-establish equilibrium shape (i.e., time for coalescence completion) increases with increasing island radius (i.e., size) R [82,83], until it becomes longer than the time required for a third island to impinge on a coalescing island pair. This point during growth corresponds to the so-called elongation transition, beyond which the film surface consists predominantly of elongated non-coalesced clusters of islands [84]. Analytical modelling, based on the droplet growth theory [85,86], and kinetic Monte Carlo simulations [87,88,89,90] suggest that, for film materials and deposition parameters for which coalescence is the dominant process during early stages of film growth (coalescence-controlled growth regime), the nominal film thickness at the elongation transition $h_{elong}$ scales with F (for the case of 3D growth) as

(7) $$h_{elong}\;\sim {\left(\frac{B}{F}\right)}^{\frac{1}{3}}$$

In Equation (7) B is the so-called coalescence strength, which is a material- and temperature-dependent constant [82,83]. Equation (7) reflects the effect of dynamic competition among island growth and coalescence on film morphological evolution. For a constant coalescence strength B, increase of F yields a larger island growth rate, such that an elongated surface morphology is attained at smaller nominal thicknesses. Conversely, an increase of B, at a constant F, promotes coalescence completion relative to island growth, thereby delaying the occurrence of elongation transition.

For a given film/substrate system, there are deposition conditions in terms of F and T, for which coalescence is not completed throughout all stages of growth (coalescence-free growth regime) [87,88,89,90]. In this case, $h_{elong}$ becomes proportional to the island-island separation distance when island density reaches $N_{sat}$ [87,88,89,90], i.e., $h_{elong}\sim N_{sat}{}^{-\frac{1}{2}}$. Using Equation (6) for 3D growth, the following expression is obtained:

(8) $$h_{elong}\sim {\left(\frac{D}{F}\right)}^{\frac{1}{7}}$$

Equation (8) represents the way by which the interplay among island nucleation and growth (in case that coalescence completion is inactive) affects the early-stage film morphology. An increase of D, for a given F, favors the growth of existing islands, at the expense of nucleation of new ones, resulting in an increase of the nominal thickness required for the onset of island-island impingement. In the opposite case, larger F, at a constant D, promotes nucleation, pushing elongation to occur at smaller nominal thicknesses.

Percolation transition and continuous-layer formation: The onset of elongation transition leads to a film surface that is predominantly covered by polycrystalline islands. The shapes of grain boundaries in these islands change continuously as result of the competition between boundary and surface energies [23], while grain boundaries can be mobile, depending on the growth temperature and the grain size [21]. These effects cause grain growth, which in combination with the kinetically controlled rate at which adatoms descend from the surface of the film to the grain boundary base (hole filling) leads to a formation of an interconnected island network (percolation transition) and eventually to a continuous film. Intuitively, it should be expected that the nominal thickness at which percolation transition occurs ($h_{perc}$) and a continuous film is formed ($h_{cont}$) are affected only by the rates of hole filling vs. out-of-plane film growth. However, the influence of initial growth stages (island nucleation, growth, and coalescence) on the values and scaling behavior of those thicknesses is very pronounced, as explained in Section 3.2.

### 3.2. Experimental Determination of Morphological Transition Thicknesses

Initial growth stages related to island nucleation are typically studied by scanning tunneling microscopy (STM) [22], which is an ideal tool for investigating morphology in epitaxial film/substrate systems, including metals deposited on oxide surfaces [1,30]. However, due to the inherent complexity of STM techniques, most studies of metal film growth on weakly-interacting substrates focus on post-nucleation morphological transitions (elongation, percolation, continuous-film formation) and their respective nominal thicknesses. The absolute value of the elongation transition thickness $h_{elong}$ for a given set of synthesis conditions reflects the degree of 3D clustering during growth, whereby larger $h_{elong}$ indicates a more pronounced 3D morphology. Concurrently, the scaling behavior of $h_{elong}$ as a function of the deposition rate F describes the relative importance of nucleation vs. coalescence for film morphological evolution [89] (see Equations (7) and (8)). The elongation transition is an intrinsically abstract concept, i.e., $h_{elong}$ is difficult to determine experimentally [91]. Hence, subsequent morphological transition thicknesses, i.e., $h_{perc}$ and $h_{cont}$, are typically measured, as these thicknesses have been shown to scale linearly with $h_{elong}$ [87,88,91].

**Figure 4:**
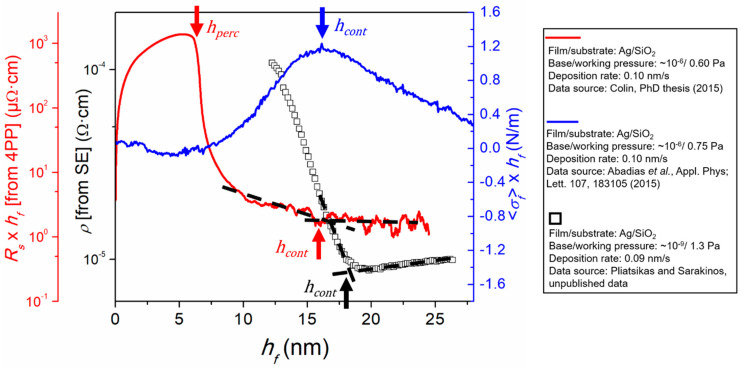
Evolution of resistivity ($\rho \sim R_{s}\times h_{f})$ and stress-thickness product $\langle \sigma_{f}\rangle \times h_{f}$ vs. nominal film thickness $h_{f}$ during growth of Ag on SiO_2_ by magnetron sputtering, as measured from in situ and real-time diagnostic tools: $R_{s}$ (from four-point-probe measurements; red solid line; data taken from [105], ρ (from spectroscopic ellipsometry (SE); hollow black squares; unpublished data by Pliatsikas and Sarakinos), and $\langle \sigma_{f}\rangle \times h_{f}$ (from multiple-beam optical stress sensor (MOSS) measurements; data taken from [73]). Growth conditions (deposition rate and base/working pressure) are indicated in the corresponding legends, while all films have been grown at room temperature. The percolation ($h_{perc}$) and continuous film formation ($h_{cont}$) thicknesses are determined by the curves as explained in the text.

The formation of an interconnected network of islands (i.e., percolation transition) leads to the onset of electrical conductivity, when a film is deposited on a substantially insulating substrate. Hence, $h_{perc}$ can be determined by measuring in situ the resistivity change of the deposited layer (using the four-point-probe technique; see Section 2.3), whereby $h_{perc}$ corresponds to the thickness at which the measured resistivity exhibits a sharp drop. An example is shown in Figure 4, which plots a $R_{s}\times h_{f}$ vs. $h_{f}$ curve from an Ag film grown by magnetron sputtering on SiO_2_ (red solid line; percolation thickness marked with a red solid arrow) [61]. With increasing film thickness beyond $h_{perc}$, the resistivity decreases further until the $R_{s}\times h_{f}$ vs. $h_{f}$ curve reaches a steady-state (marked by the intersection of the dashed lines and indicated with a red solid arrow). Multiple studies [61,62,92,93,94], which have combined in situ growth monitoring and ex situ morphology and structure characterization, have shown that the thickness at which steady-state film resistivity is established corresponds to $h_{cont}$. An alternative approach for determining film resistivity is indirectly by SE using the Drude model, as explained in Section 2.4. A resistivity ρ vs. $h_{f}$ curve, determined by SE, for Ag grown by magnetron sputtering on SiO_2_ is also plotted in Figure 4 (black hollow squares), and $h_{cont}$ (i.e., onset of steady-state behavior) is marked with a black solid arrow at the intersection between the two dashed solid lines used as a guide to the eye. We note here that accuracy of the Drude model close to the onset of conductivity is limited, hence $h_{perc}$ cannot be determined with precision using SE [62].

Stress evolution is also closely connected with the film formation stages [95]. Figure 4 plots the stress-thickness $\langle \sigma_{f}\rangle \times h_{f}$ vs. $h_{f}$ curve of an Ag layer grown by magnetron sputtering on SiO_2_ (blue solid line) [61]. The curve exhibits the typical compressive-tensile-compressive (CTC) stress evolution for films grown at conditions of high atomic mobility on weakly-interacting substrates [67,70,95]. The origin of the different stress stages has been the focus of extensive experimental and theoretical works in the literature. It is widely accepted that the first compressive stage corresponds to the nucleation of isolated islands [95,96], while their coalescence leads to tensile stress formation (attractive forces) due to elastic strain upon impingement of neighboring surfaces (similar to a zipping process, see [97,98]), the driving force being the reduction in surface/interface energy upon formation of grain boundary between coalescing islands pairs. As coalescence progresses, the film continues to develop tensile stress up to an observable maximum, which has been shown to coincide with the formation of a continuous layer [73,99]. Therefore, the stress monitoring during thin film growth using MOSS allows the straightforward determination of $h_{cont}$ from the position of the tensile peak maximum, as indicated by the blue arrow in Figure 4. The underlying mechanisms for the origin of the compressive stress in the continuous film growth regime are still the subject of debate [100,101,102,103,104], and will not be further discussed here, as they fall outside the focus of the present review paper. We also note that the differences in the morphological transition thicknesses established by the curves in Figure 4 reflect differences in growth kinetics, as determined by the deposition conditions (e.g., deposition rate, temperature, base and working pressure). A more detailed discussion on this aspect is provided in Section 3.3.

### 3.3. The Effect of Growth Kinetics on Film Morphological Evolution

#### 3.3.1. Influence of Material Intrinsic Mobility

**Figure 5:**
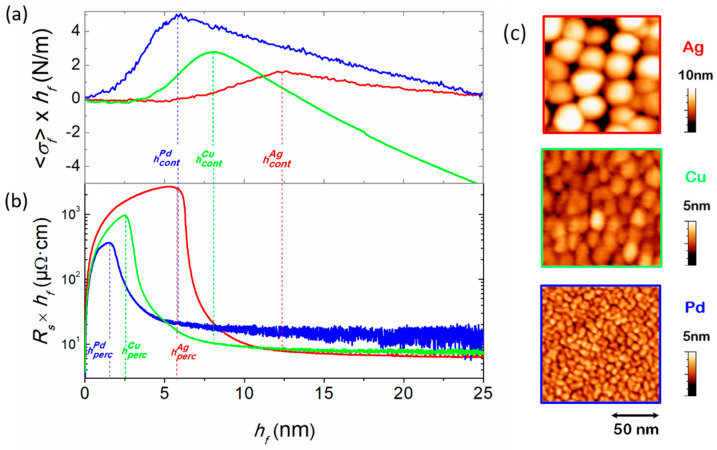
Real-time evolution of (**a**) $\langle \sigma_{f}\rangle \times h_{f}$ and (**b**) $R_{s}\times h_{f}$ vs. $h_{f}$ for sputter-deposited Ag (red solid line), Cu (green solid line) and Pd (blue solid line) films on SiO_2_ at *T* = 300 K. Morphological transition thicknesses $h_{perc}$ and $h_{cont}$ are indicated on the respective curves with vertical dashed lines of the same color. (**c**) Atomic force microscopy (tapping mode) images (125 × 125 nm^2^) showing the surface morphology of Ag, Cu and Pd films with $h_{f}$~3 nm. More information on the growth conditions for each data set is provided in Table 1. Data taken from [71,78].

In the present section, we demonstrate the ability of the in situ and real-time techniques described in Section 2, to establish the effect of growth kinetics, as determined by synthesis conditions and intrinsic material properties, on the degree of 3D clustering in weakly-interacting film/substrate systems. We start by examining the evolution of $\langle \sigma_{f}\rangle \times h_{f}$ (Figure 5a) and $R_{s}\times h_{f}$ (obtained from four-point-probe measurements; Figure 5b) during growth of Ag, Cu, and Pd on SiO_2_, at otherwise identical deposition conditions (see Table 1 for deposition conditions). The $\langle \sigma_{f}\rangle \times h_{f}$ curves exhibit the characteristic CTC stress evolution as a function of $h_{f}$, from which the continuous film formation thickness $h_{cont}$ (i.e., tensile peak maximum) is determined for each metal. The $R_{s}\times h_{f}$ curves show the abrupt drop at $h_{perc}$, while the onset of a steady-state signifies $h_{cont}$ (all transition thicknesses are marked with vertical dashed lines in the respective curves in Figure 5). The values of $h_{perc}$ (from $R_{s}\times h_{f}$ vs. $h_{f}$ curves) and $h_{cont}$ (from $\langle \sigma_{f}\rangle \times h_{f}$ vs. $h_{f}$ curves) are listed in Table 1, where it is seen that Ag exhibits the largest values for both quantities ($h_{perc}$ = 5.9 nm and $h_{cont}$ = 12.4 nm), followed by Cu ($h_{perc}$ = 2.6 nm and $h_{cont}$ = 8.2 nm), while Pd has the smallest values with $h_{perc}$ = 1.7 nm and $h_{cont}$ = 5.9 nm. These differences in morphological transition thicknesses indicate that Ag grows in the most pronounced 3D fashion (among the three metals), while Pd exhibits the flattest morphology, as confirmed by ex situ studies of the surface topography of the three metals, see Figure 5c. These findings are also consistent with the TEM observations of Figure 3, where Cu islands form more elongated structures and percolate at lower film thickness compared to Ag. Concurrently, the three metals have distinctly different melting points $T_{m}$, so that their homologous temperatures $T_{h}=T/T_{m}$ (T is the deposition temperature that is 300 K, common for all metals) are 0.24 (Ag), 0.22 (Cu), and 0.16 (Pd) (also listed in Table 1). Atomic mobility, in a first approximation, scales with $T_{h}$ [21], i.e., Ag exhibits the largest mobility. This allows Ag to: (i) diffuse longer distances on the substrate and self-assemble into larger and fewer nuclei; (ii) exhibit more pronounced upward diffusion from the base to the top of atomic islands [106,107]; and (iii) diffuse faster on Ag islands so the coalescence completion is promoted [108]. All the aforementioned effects favor 3D growth morphology, and thereby, yield the largest $h_{perc}$ and $h_{cont}$ values. Using the same argument, we can identify the reason for the smallest $h_{perc}$ and $h_{cont}$ values observed for Pd (i.e., less pronounced 3D morphology) to the smaller atomic mobility. Similar findings were reported by Abermann et al. during thermal evaporation of Ag, Au and Cu films [109].

Atomic mobility is not the sole factor that governs the stress and morphological evolutions. Structure formation (crystalline phase) is another aspect that needs to be investigated. In this example, the three metals crystallize in their fcc structure with (111) preferred orientation, but there are scenarios in which nucleation of a specific crystallographic phase is influenced by interfacial effects. This will be discussed in Section 4.1. Another aspect related to Pd is its reactivity with the Si substrate, which may increase interaction strength, change interface chemistry, and suppress 3D growth. This issue is further examined in Section 4.2.

#### 3.3.2. Influence of Deposition Rate F and Temperature T

**Figure 6:**
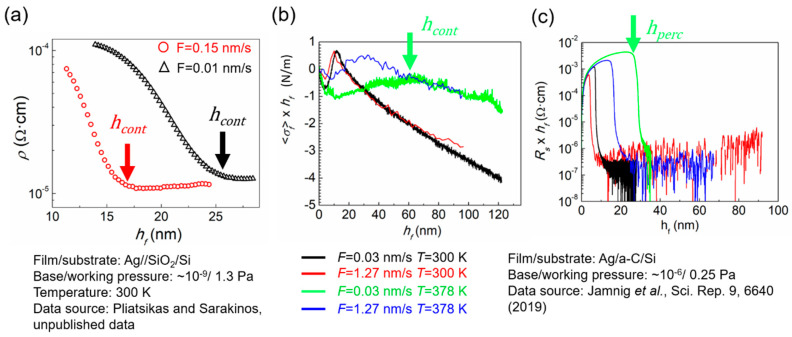
(**a**) ρ vs. $h_{f}$ curves, extracted from in situ spectroscopic ellipsometry, during room-temperature (300 K) magnetron-sputter deposition of Ag films on SiO_2_/Si substrates at two different deposition rates F of 0.15 (red circles) and 0.01 nm/s (black triangles). The position of $h_{cont}$ on the curves is indicated by solid arrows (unpublished data by Pliatsikas and Sarakinos) (**b**) $\langle \sigma_{f}\rangle \times h_{f}$ vs. $h_{f}$ curves, extracted from in situ substrate curvature measurements, during deposition of Ag films on a-C/Si substrates at two values of F (0.03 and 1.27 nm/s) and T (300 and 378 K). (**c**) $R_{s}\times h_{f}$ vs. $h_{f}$ curves, extracted from in situ four-point probe measurements, during deposition of Ag films on a-C/Si substrates at two values of F (0.03 and 1.27 nm/s) and T (300 and 378 K). The positions of $h_{cont}$ and $h_{perc}$ on the curves at T=378 K and F = 0.03 nm/s are indicated by solid arrows. Data in (**b**) and (**c**) are taken from [61].

As explained in Section 3.1, growth kinetics and the resulting film morphology can be affected and controlled by varying the deposition rate F (through change in the sputtering power) and the deposition temperature T. This is demonstrated in Figure 6, which plots ρ vs. $h_{f}$ curves, extracted from in situ SE, during room-temperature (300 K) magnetron-sputter deposition of Ag films on SiO_2_/Si substrates (Figure 6a) at two different deposition rates F of 0.15 (red circles) and 0.01 nm/s (black triangles). The data show that an increase of F yields a decrease in $h_{cont}$ (indicated by vertical solid arrows). The ability of the in situ and real time techniques described in Section 2 to establish changes in morphological transition thicknesses ($h_{perc}$ and $h_{cont}$) as a function of deposition conditions is further illustrated by the $\langle \sigma_{f}\rangle \times h_{f}$ and $R_{s}\times h_{f}$ vs. $h_{f}$ curves (Figure 6b,c, respectively). These curves have been recorded during deposition of Ag films on a-C/Si substrates at various values of F and T [61], whereby decrease (increase) of F(T) leads to higher values of $h_{perc}$ and $h_{cont}$.

**Figure 7:**
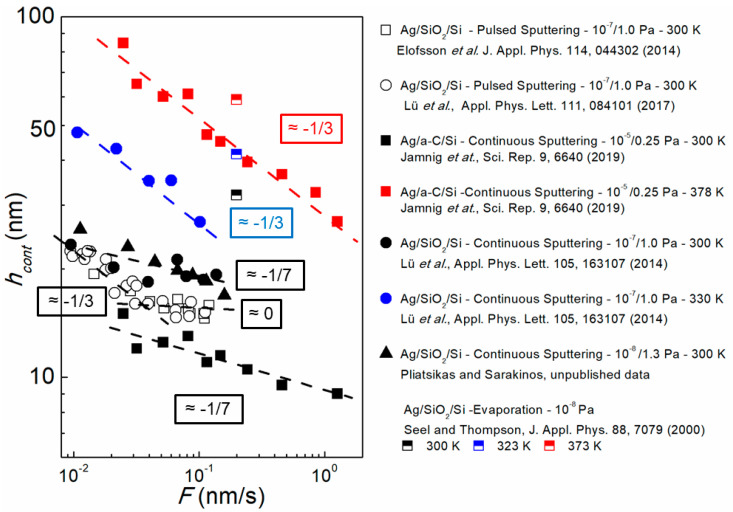
$h_{cont}$ vs. F (log–log scale) at various values of *T* in the range 300 to 378 K, during growth of Ag on a-C and SiO_2_ substrates by continuous and pulsed magnetron sputtering (data from [61,62,89,91]) and evaporation (data from [99]). The dashed lines are guides to the eye indicating the $h_{cont}$ vs. F slopes (provided next to each line) for selected set of data. More information on the growth conditions for each data set, including base and working pressure, is provided in the respective legends.

To further discuss the effect of deposition conditions on film morphological evolution, we plot in Figure 7 $h_{cont}$ (for Ag layers grown on SiO_2_ and a-C substrates determined by substrate curvature and ellipsometry measurements as those presented in Figure 6) as a function of F (log–log scale) for various T values. Data are extracted for films deposited by continuous and pulsed magnetron sputtering (filled and hollow symbols, respectively) [61,62,89,91], and by evaporation (half-filled symbols) [99] at different conditions with regards to base and working (i.e., Ar gas in case of sputtering) pressures, as indicated in the respective legends in Figure 7. The first observation (similar to the data in Figure 6) is that, for all conditions and deposition techniques reported in Figure 7, the magnitude of $h_{cont}$ decreases with F, which indicates that 2D growth morphology is favored by larger arrival rates of vapor at the film growth front. As explained in detail in Section 3.1, this can be attributed to either increase of island number density or delay of cluster reshaping during coalescence. In addition, we see that the $h_{cont}$ vs. F data exhibit a power-law dependence (straight lines in log–log scale), in accordance to Equations (7) and (8). Closer inspection of the data reveals that room-temperature (*T* = 300 K) continuous magnetron-sputter-deposition (black filled symbols) yield negative slopes of 1/7, which is indicative of coalescence-free growth (Equation (8)). Increase of temperature to T = 330 K (blue filled circles) and *T* = 378 K (red filled squares) results to larger $h_{cont}$ values, i.e., tendency toward 3D morphology is enhanced, as atomic mobility, nucleation, and island reshaping are promoted. Moreover, larger growth temperatures lead to larger negative $h_{cont}$ vs. F slope magnitudes (~1/3), i.e., morphology evolves closer to the coalescence-controlled growth (Equation (7)). By establishing the growth regimes and using $h_{cont}$ data for multiple F and T values, rates of atomic-scale processes that control island nucleation, growth, and coalescence can be estimated [61,91]. Another point of interest in the data plotted in Figure 7, is that for pulsed sputtering (hollow symbols) $h_{cont}$ exhibits a complex scaling dependence on F with $h_{cont}$ vs. F slope changing from ~−1/3 to ~0 with increasing deposition rate. This is a signature of multiple nucleation regimes encountered in pulsed deposition, as explained by Jensen and Niemeyer [110] and Lü et al. [90].

**Figure 8:**
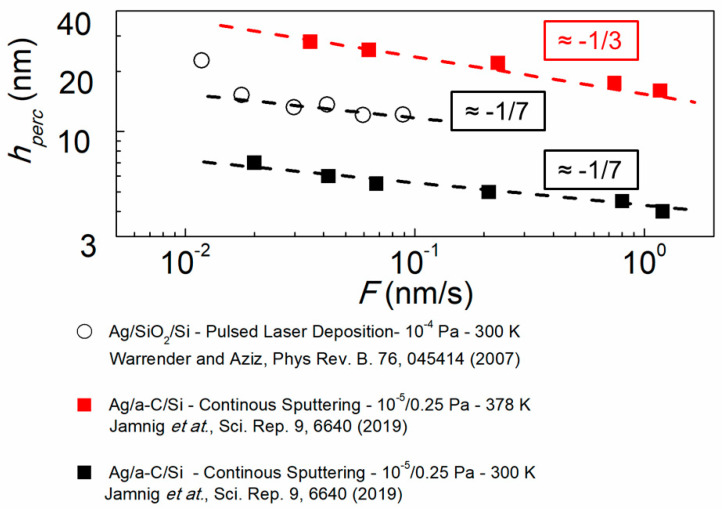
Percolation thickness $h_{perc}$ vs. deposition rate F (log–log scale) at growth temperatures *T* = 300 and 378 K, during the growth of Ag on a-C substrates by continuous sputtering (data from [61]), and pulsed laser deposition (data from [111]). The dashed lines are guides to the eye indicating the $h_{perc}$ vs. F slopes (provided next to each line) for selected set of data. More information on the growth conditions for each data set, including base and working pressure, is provided in the respective legends.

Besides $h_{cont}$, we also plot in log–log scale in Figure 8 $h_{perc}$ vs. F ($h_{perc}$ determined by four-point-probe measurements as shown in Figure 6) for Ag films grown on a-C using continuous magnetron sputtering, at *T* = 300 K (black filled squares) and *T* = 378 K (red filled squares) [61]. We see that the $h_{perc}$ vs. F line slopes are consistent with those in Figure 7 ($h_{cont}$ vs. F) for both low and high deposition temperatures, while the ratio $h_{cont}$/$h_{perc}$ takes a value of ~2, irrespective of the growth temperature. This indicates that the morphology set at the early stages of island nucleation and coalescence remains consistent until continuous layer formation. Figure 8 also presents $h_{perc}$ vs. F data obtained during the growth of Ag on SiO_2_ by pulsed layer deposition (hollow circles) [111]. This set of data exhibits a negative slope of ~1/7 in the F range 10^−2^ to 10^−1^ nm/s with a tendency for larger slope for $F<10^{-2}\;\mathrm{nm}/s$, in qualitative agreement with the $h_{cont}$ vs. F lines for pulsed magnetron sputtering in Figure 7.

The data presented in Figure 7 and Figure 8 highlight the possibility to fine tune the thickness at which the metallic film becomes electrically conductive or continuous by appropriate control of the vapor flux rate or substrate temperature. For instance, in the case of Ag films, $h_{cont}$ can be varied in a relatively broad range from ~10 nm at room temperature and F = 1 nm/s up to ~50 nm at *T* = 330 K and F = 0.01 nm/s. This strategy has important implications for practical applications since the control of the early growth stages is decisive in achieving the desired physical attributes which critically depend on the film microstructure.

#### 3.3.3. Other Factors Influencing Film Morphological Evolution

Base pressure: As explained in Section 2.1, data on Ag and Cu were obtained from depositions in vacuum chambers with different base pressures. It can be seen in Figure 7 that $h_{cont}$ is larger for Ag films sputter-deposited in better vacuum conditions (i.e., lower base pressure). This trend is particularly salient at low deposition rates (compare filled triangles vs. filled circles which correspond to base pressures 10^−8^ and 10^−7^ Pa, respectively), and it can be explained by the influence of residual gas contaminants (typically H_2_O, O_2_, CO) which become adsorbed on the growth surface and act as preferred nucleation sites. This effect has been discussed in the early works by Abermann and Koch [109], who have shown that the tensile peak maximum of the $\langle \sigma_{f}\rangle \times h_{f}$ vs. $h_{f}$ curves recorded for a series of thermally evaporated metal films was shifted to lower values under poorer vacuum conditions. This is most often accompanied by the development of more tensile stress in the continuous film regime, as confirmed in our recent study on sputter-deposited Cu films [112].

Energy of film-forming species: Figure 7 shows that sputter-deposition yields systematically smaller $h_{cont}$ at all temperatures (for a given deposition rate) compared to evaporated films. This can be attributed to the fact that the film growth front during sputtering is exposed to a more energetic (vs. thermalized for evaporation) vapor flux [63,113], which is known to enhance nucleation center density, and thereby promote flat morphology via defect formation and cluster dissociation [22]. Such effects are consistent with earlier reports by Grachev et al. [49] who used in situ and real-time surface differential reflectance spectroscopy and established that the in-plane size of Ag islands formed during sputter-deposition is 3 to 4 times larger compared to Ag islands produced by thermal evaporation. Moreover, for sputter-deposited Cu films, $h_{cont}$ has been shown to decrease from ~16 nm to ~9 nm when decreasing the Ar working pressure from 2 to 0.05 Pa [114], confirming the trend that a larger flux of energetic species to the film growth front (e.g., achieved by decreasing working pressure during sputtering) promotes in-plane island growth and leads to earlier onset of island impingement. This is consistent with the data reported in Figure 7 which also show that the working pressure has influence on the thickness at which Ag films become continuous, with larger $h_{cont}$ values reached at higher Ar working pressure at which energetic sputtered particles experience more collisions in the gas phase, and hence arrive at the substrate with lower kinetic energy and create fewer surface defects. At fixed working pressure, similar trends can be expected by increasing the target-to-substrate distance.

Besides changing the working pressure, the energetic bombardment during growth can be tuned by the application of a negative bias voltage to the substrate. In conventional direct current magnetron discharges, the ionization degree of the sputtered material is very low (1–2%) and working gas ions are the main ionized species in the plasma, so that the application of a bias voltage may result in generation of point defects, larger compressive stress values [113], and decreased charge carrier mobility. However, in high power impulse magnetron sputtering (HiPIMS) discharges, a large fraction of sputtered atoms, up to 50–80%, gets ionized [115], so that applying a bias voltage can be advantageously used to tailor the film microstructure by controlling the energy of film-forming species. Cemin et al. [116] have reported that the thickness at which Cu films are continuous could be increased from $h_{cont}$ ~7 to ~12 nm by increasing the bias from 0 to 130 V, due to enhanced adatom mobility. This results in the formation of Cu films with larger grain size, reduced compressive stress, and higher electrical conductivity [116,117].

Surfactants: The morphology of high-mobility metal films (e.g., Ag) can be further manipulated, for a given set of deposition conditions with regards to F and T, by using minority gaseous (N_2_, O_2_) or metallic (e.g., Al, Cu, and early transition metals) species to act as wetting agents. Such species can be either deposited as seed layers to change the energy and kinetics at the film/substrate interface and affect nucleation [118], or co-deposited at the film growth front, which modifies the island coalescence dynamics [17,18,79,119,120,121,122] as well as stress response [123,124]. In this framework, in situ and real-time growth monitoring methodologies provide insights for selective deployment of wetting agents at key formation stages such that film morphology can be manipulated without compromising other physical properties of the film/substrate system [79,119,120].

### 3.4. Studies of Discontinuous Ag-Layer Morphology

Section 3.3 has demonstrated the way by which in situ and real-time techniques can be used to determine growth transition thicknesses ($h_{perc}$ and $h_{cont}$) and their correlation with the dynamics of film morphological evolution. In the present section, we showcase the use of in situ and real-time SE for studying the growth of discontinuous layers and establish the correlation with percolated and continuous films. To this purpose, we use Ag/SiO_2_ as a model system (Ag is deposited by continuous magnetron sputtering at a base pressure of 10^−8^ Pa and at F=0.1 nm/s) and investigate the effect of deposition temperature (*T* = 300 and 500 K).

**Figure 9:**
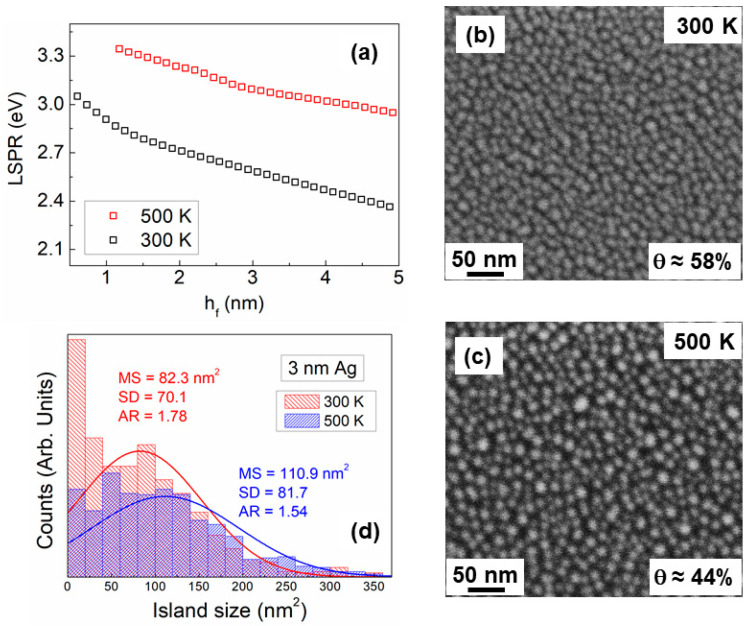
Morphological studies of discontinuous Ag layers deposited by magnetron sputtering on SiO_2_ substrates (base pressure 10^−8^ Pa; working pressure 1.3 Pa; *F* = 0.1 nm/s) at two deposition temperatures of *T* = 300 and 500 K (unpublished data by Pliatsikas and Sarakinos). (**a**) Evolution of localized surface plasmon resonance (LSPR) vs. nominal film thickness, as determined from in situ and real-time SE. Plan-view SEM images of 3 nm thick films are shown in (**b**) for *T* = 300 K and (**c**) for *T* = 500 K. (**d**) Quantitative analysis of SEM images with regards to island size distribution, mean island size MS, size standard deviation SD, and in-plane aspect ratio AR.

Analysis of SE data of electrically conductive layers, using the Drude model and the methodologies explained in Section 2.4, showed that $h_{cont}$ increases from ~26 to >80 nm, when *T* is increased from 300 to 500 K, i.e., higher temperature promotes 3D (in agreement with the data in Figure 7). Early growth stages of discontinuous layers are investigated by establishing the evolution of LSPR (ellipsometric data analyzed using Lorentz model as explained in Section 2.4) as a function of $h_{f}$. The results are shown in Figure 9a, where it is seen that for both deposition temperatures the energy of LSPR decreases (from ~3.3 to ~2.4 eV, i.e., is red-shifted) with increasing $h_{f}$. This trend signifies that the mean island size increases and island separation shrinks (i.e., larger fraction of the substrate is covered) as more material is deposited [55,79,119]. Concurrently, we observe that the LSPR vs. $h_{f}$ slope for *T* = 300 K is steeper than its 500 K counterpart, which means that increase of deposition temperature promotes out-of-plane island growth yielding a lower substrate coverage for a given nominal thickness [79,119]. These differences are confirmed by plan-view scanning electron microscopy (SEM) images of 3 nm thick films at the two growth temperatures (Figure 9b,c), respectively). At *T* = 300 K, the film surface features islands that are partially elongated and interconnected, indicating that incomplete coalescence has started, while the substrate coverage is θ = 58%. In contrast, at *T* = 500 K, islands are clearly more circular and larger (i.e., island reshaping is completed during coalescence), and θ = 44%. The qualitative picture and its consistency with the LSPR evolution is further supported by quantitative analysis of the SEM images with regards to the island size distribution and the mean island in-plane aspect ratio (AR). Data (presented in Figure 9d) show that the mean island size MS increases from ~82 to 110 nm^2^ upon temperature increase from 300 to 500 K, while AR decreases from ~1.8 to 1.5 for the same change in temperature (AR = 1 for circular islands).

## 4. Interface Reactivity and Structure Formation

The present section highlights the use of the in situ and real-time diagnostic tools presented in Section 2 for studying more complex scenarios of thin film growth involving chemical reactivity between the deposited metal and the underlying substrate/layer. This is typically the case at the metal/Si interface, where silicide layers usually form by solid-state reaction during annealing [26]. Their formation can also be driven by dynamic processes during film growth, such as segregation or energetic bombardment-induced intermixing. To this purpose, we present and discuss data with regards to growth of bcc transition metals (Mo, Ta, Fe) on Si, which showcase the ability to study amorphous-to crystalline transitions, as well as formation of different crystallographic allotropes during initial film-formation stages. We also demonstrate the potential of combining real-time wafer curvature and resistivity techniques to identify complex interface chemical reactions during growth of Pd on Si and Ge and correlate them to the structure formation during subsequent metal growth.

### 4.1. Growth of Fe, Mo, and Ta on a-Si

#### 4.1.1. Structure and Phase Formation

We report here the data on real-time monitoring—using MOSS and resistivity diagnostics—of the growth evolution of sputter-deposited Fe, Mo, and Ta films (bcc crystal structure), on Si substrates covered with a 9 nm thick a-Si layer. The a-Si overlayer was sputter-deposited in the same vacuum chamber prior to growth of the metal layer. This methodology provides the same starting growth surface for all experiments and minimizes the influence of oxygen contamination on film growth. Depositions were performed at room temperature (300 K) and at relatively low Ar working pressure (0.24 Pa). Similar deposition rates, as given in Table 2, were employed for all metal layers by controlling the power applied to the respective magnetron sources, such as to rule out possible effects of vapor flux rate *F* on the observed growth evolution (as discussed in Section 3). At *T* = 300 K, the respective homologous temperatures $T_{h}$ of the three metals are 0.16 (Fe), 0.10 (Mo), and 0.09 (Ta). Based on this, it is expected that Mo and Ta grow at conditions of limited atomic mobility ($T_{h}$~0.1), and hence exhibit zone-I type (i.e., underdense) microstructure [63,125,126]. However, atomic mobility is enhanced by energetic bombardment during sputter-deposition at low working pressure [63,113], resulting in fully-dense films, as confirmed by ex situ XRR [127,128].

**Figure 10:**
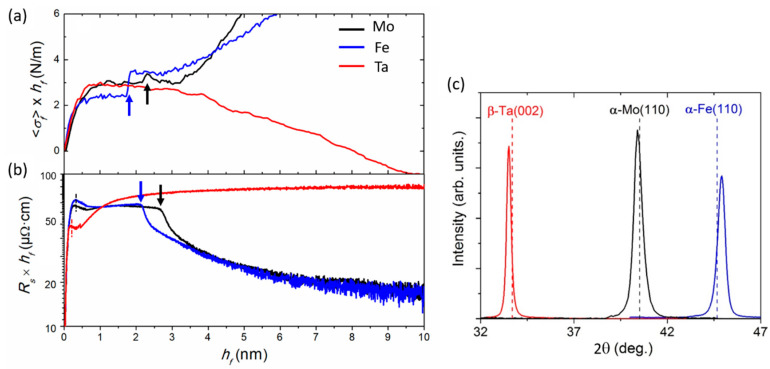
Real-time evolution of (**a**) $\langle \sigma_{f}\rangle \times h_{f}$ and (**b**) $R_{s}\times h_{f}$ curves vs. film thickness $h_{f}$ during the early growth stages of Fe, Mo, and Ta films on a-Si at *T* = 300 K (deposition conditions are indicated in Table 2). The arrows in (**a**) and (**b**) indicate the onset of film crystallization for Fe and Mo. (**c**) XRD patterns recorded in Bragg–Brentano configuration from ~60 nm thick Fe, Mo, and Ta films using Cu Kα radiation (λ = 0.15418 nm). MOSS data are taken from [105,128,134].

Figure 10a,b shows the evolution of $\langle \sigma_{f}\rangle \times h_{f}$ and $R_{s}\times h_{f}$ vs. $h_{f}$ during the early growth stages of the three metals, up to $h_{f}$ = 10 nm. Fe and Mo show similar trends of tensile stress build-up and decrease in resistivities upon film thickening. In contrast, the behavior of Ta is distinctly different: the incremental stress becomes compressive for $h_{f}$ ≥ 2 nm and the film retains a high electrical resistivity. Ex situ XRD investigations of the crystal structure on thicker films (~60 nm thick) uncover that, while Mo and Fe crystallize in their equilibrium bcc structure, the β-Ta allotrope (tetragonal structure [129,130]) forms in sputter-deposited Ta films [128], as shown in Figure 10c. Hence, the observed differences in the early-stage of stress and resistivity evolutions can be understood as fingerprints of nucleation and growth of different crystallographic phases.

Another striking observation in the curves corresponding to Mo and Fe films presented in Figure 10a,b is the occurrence of transient features which manifest themselves as a concomitant tensile jump and resistivity drop (indicated by arrows). These abrupt variations occur at $h_{f}$ = 1.9–2.1 nm and $h_{f}$ = 2.2–2.6 nm for Fe and Mo, respectively, while they are not observed during growth of Ta. Note that the thickness values of these transient features differ by ±0.2 nm between the two in situ techniques since the measurements were not performed simultaneously, but have the same physical origins, as explained in the following. In the case of Ta, no transient feature is observed in the $\langle \sigma_{f}\rangle \times h_{f}$ and $R_{s}\times h_{f}$ vs. $h_{f}$ curves, and the β-Ta phase is formed, which is intrinsically more resistive (typical resistivity of 170 µΩ·cm compared to 25–30 µΩ·cm for bcc-Ta [128]) and of lower crystal symmetry. It was proposed that this phase is stabilized by minimization of interface energies [128], although other authors argued that phase formation is governed by the presence of TaO*_x_* interlayer and template (epitaxial) growth [131].

In the case of Mo/a-Si, the transient stress feature observed at around 2.2 nm (a value consistent with literature reports [132]) was shown to correspond to the onset of an amorphous-to-crystalline (a–c) phase transition [133,134], which was unambiguously established recently by coupling in situ MOSS and XRD measurements [135]. The structure formation pathway in the case of Mo deposition on a-Si can be summarized as follows: (i) Mo atoms react with Si surface atoms, forming an amorphous silicide interfacial layer over a thickness of ~2 nm [132]; (ii) at a critical thickness of 2.2 ± 0.1 nm, the amorphous layer crystallizes into bcc Mo. The a–c transition results in a volume contraction (film densification) and structural ordering, which are reflected by the formation of tensile stress and decrease in film resistivity (Figure 10a,b), respectively). Due to the observed similarities among the real-time curves of Fe and Mo, we argue that the structure formation process during growth of Fe on a-Si obeys the same scenario (as for Mo). Steiner et al. [136] reported the formation of an interfacial amorphous layer during growth of Fe film on B for $h_{f}$ ≤ 3 nm. Interestingly, a drop in electrical resistance was evidenced at $h_{f}$~3 nm, likely imputable to crystallization, although the authors for [136] did not explicitly draw this conclusion.

#### 4.1.2. Early Growth Morphology and Interface Stress

In the present section, we focus on the incipient growth stages ($h_{f}$ < 2 nm) of Mo, Ta, and Fe films, as evidenced by the data presented in Figure 10a,b. The evolution of the $R_{s}\times h_{f}$ vs. $h_{f}$ curves shows an initial drop below 0.5 nm (indicated by dashed lines in (Figure 10b), which can be attributed to film percolation. Compared to the $h_{perc}$ values reported in Section 3 for Ag, Cu, and Pd films deposited on weakly-interacting substrates, the percolation thickness found for Fe, Mo, and Ta are extremely small, around 0.3–0.4 nm (see Table 2), which corresponds to the deposition of ~2 monolayers of film materials. This clearly shows that Mo, Ta, and Fe exhibit a pronounced 2D (i.e., wetting) growth morphology on a-Si, further supported by AFM imaging of layers with thicknesses in the range of 5 nm [71].

**Figure 11:**
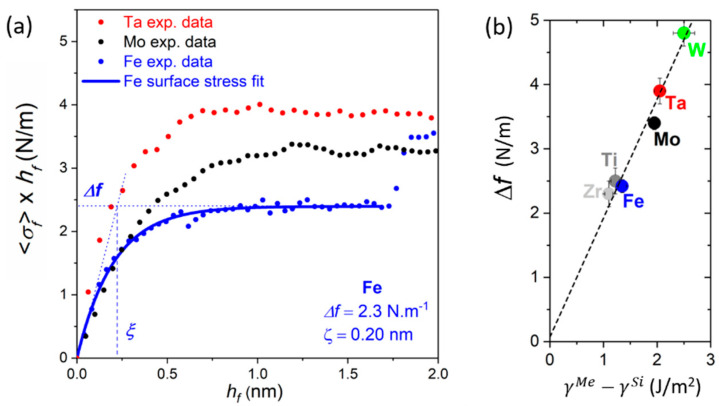
(**a**) Enlarged view of the stress-thickness evolution at the Me/a-Si interface during growth of Fe, Mo, and Ta on a-Si. An analytical model, Equation (9), is used to fit experimental data (see solid line for the case of Fe) to extract Δf and ξ. (**b**) Plot of Δf vs. Δγ for bcc Fe, Mo, Ta and W (data obtained from present work and Refs. [71,105]) and hcp Ti and Zr (unpublished data by Abadias) deposited on a-Si. and. Surface energies $\gamma^{Me}$ and $\gamma^{Si}$ are taken from [139].

Concomitantly, the $\langle \sigma_{f}\rangle \times h_{f}$ curves (plotted for 0 $\leq h_{f}\leq$ 2 nm in Figure 11a) show the formation of tensile stress, an opposite behavior to what is observed in Figure 3 for metal films on weakly-interacting substrates. The tensile stress levels off until a plateau is reached, which can be understood by the change in surface/interface stress Δf due to the progressive coverage of the a-Si surface (surface energy γ^Si^) by a metal (Me) deposit (γ^Me^ > γ^Si^). The quantities *f* and γ are of the same order of magnitude, and for an isotropic solid surface are related through the expression $f=\gamma +\frac{\partial \gamma }{\partial \varepsilon }$, where ε is the elastic strain within the interface plane [137,138]. Then, the asymptotic behavior of the $\langle \sigma_{f}\rangle \times h_{f}$ vs. $h_{f}$ curves in Figure 11a can be fitted by an exponential interaction term, following the approach proposed by Müller and Thomas [138], according to

(9) $$\langle \sigma_{f}\rangle \times h_{f}=\Delta f\;\left(1-e^{-h_{f}/\xi }\right)$$

where $\Delta f=f^{Me}+f^{int}-f^{Si}$ is the change in surface/interface stress during deposition of metal (Me) on a-Si, and ξ is a characteristic interaction length that accounts for the finite extension of the interface stress contribution. Δf and ξ are extracted by fitting Equation (9) to the MOSS data and values for Fe, Mo, and Ta are reported in Table 2. An example of the fit is shown in Figure 11a for the case of Fe. It is seen that the ξ values are of the same order of magnitude as $h_{perc}$ determined from resistivity measurements. Moreover, ξ increases with increasing atomic number of the metal, which may be attributed to larger interface spread (intermixing) due to more intense energetic bombardment. In the case of the Mo/a-Si interface, Reinink et al. [132] confirmed the formation of an interfacial MoSi_x_ alloy from in situ low energy ion scattering analysis, and concluded that the tensile stress was due to compound formation, rather than a change in surface stress. Nevertheless, as seen in Figure 11b, the derived Δf values are found to scale with the change in surface energy, $\Delta \gamma =\gamma^{Me}-\gamma^{Si}$, for different metals with either bcc (present work and from [71]) or hcp structure (unpublished data by Abadias). This suggests that the early growth stages of metals on a-Si are dominated by interfacial effects.

### 4.2. Effect of Interface Reactivity on Morphological Evolution

**Figure 12:**
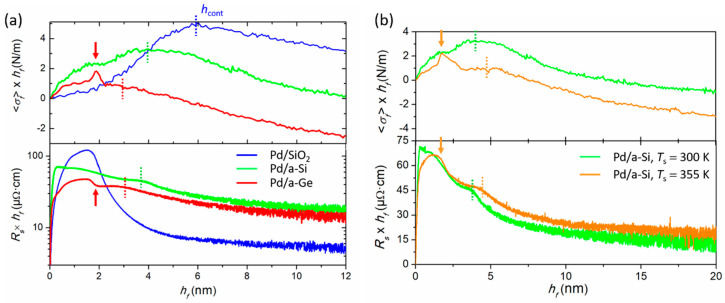
Real-time evolution of $\langle \sigma_{f}\rangle \times h_{f}$ and $R_{s}\times h_{f}$ curves vs. $h_{f}$ during sputter-deposition of Pd films on (**a**) SiO_2_, a-Si and a-Ge at *T* = 300 K and (**b**) a-Si at *T* = 300 and 355 K. The curves exhibit distinct features at critical transition thickness, marked by arrows and vertical dashed lines (for interpretation, see text).

In order to demonstrate the importance of interface reactivity on film nucleation and growth, we compare here the morphological evolution of Pd layers deposited on different substrates: SiO_2_, a-Si and a-Ge. Figure 12a,b show the $\langle \sigma_{f}\rangle \times h_{f}$ and $R_{s}\times h_{f}$ vs. $h_{f}$ real-time data collected for Pd films deposited at *T* = 300 K and *T* = 355 K, respectively. The blue curves correspond to the growth of Pd on SiO_2_, as already described in Section 3.3.1, and serve as reference. Overall, we notice that the curves corresponding to growth on a-Si and a-Ge are more complex than those for growth on SiO_2_, especially for $h_{f}<5\;\mathrm{nm}$, where the following features are observed: (i) a tensile stress is clearly formed from the beginning of growth, followed by a transient peak at ~2 nm (clearly visible on a-Ge at *T* = 300 K and a-Si at *T* = 355 K, see arrow, and smeared out in the case of a-Si at *T* = 300 K); (ii) the $R_{s}\times h_{f}$ vs. $h_{f}$ curve exhibits two consecutive drops at $h_{f}$ ~ 2 nm (marked by arrow) and below 4 nm (indicated by dashed lines). The evolution of the Pd/a-Si and Pd/a-Ge curves in Figure 12a for $h_{f}>5\;\mathrm{nm}$ is reminiscent to what is observed for the growth of Pd on SiO_2_ beyond $h_{cont}$, i.e., a compressive stress regime is established for $h_{f}>h_{cont}$, accompanied by a continuous decrease of the film resistivity. It can be noted, however, that the $R_{s}\times h_{f}$ values for Pd films deposited on a-Si and a-Ge are higher than that of the Pd film deposited on SiO_2_, while the magnitude of the incremental stress remains similar. For the Pd/a-Si system, the overall stress and resistivity evolutions are similar between *T* = 300 K and *T* = 355 K (Figure 12b). Differences in the stress development were found at higher temperatures due to higher interface reactivity [105], but will not be discussed here.

**Figure 13:**
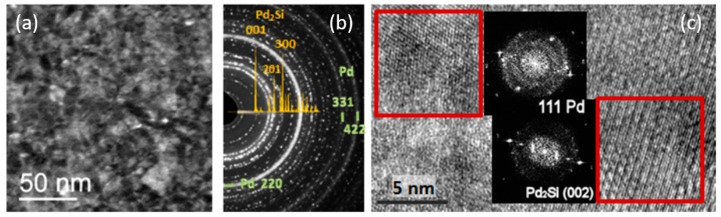
Plan-view TEM investigation of a 9 nm thick Pd film deposited on a-Si. (**a**) STEM-HAADF image revealing the grain contrast in the thin film. (**b**) selected area electron diffraction pattern showing the coexistence of polycrystalline Pd_2_Si and 111 fiber-textured Pd grains. (**c**) High resolution image; Fourier transform on selected grains are consistent either with fcc Pd or hexagonal Pd_2_Si lattice planes.

The $\langle \sigma_{f}\rangle \times h_{f}$ vs. $h_{f}$ evolution at the Pd/a-Si and Pd/a-Ge interface exhibit the same asymptotic tensile behavior as observed during growth of Fe, Mo, and Ta on a-Si (see Figure 11a), suggesting the formation of an intermixed layer. It is well known that Pd atoms react with Si to form a silicide compound with composition close to Pd_2_Si, usually obtained after thermal annealing [140,141,142,143]. The silicide formation is accompanied by the build-up of stress, being either tensile or compressive depending on the diffusion process [26,141,144]. Only few reports have studied the growth of Pd/a-Si at room temperature [140,143]. Ex situ XRD and TEM characterization performed on the Pd/a-Si system confirm the formation of a silicide compound Pd_2_Si [105]. Figure 13a,c shows plan-view TEM micrographs obtained from a 9 nm thick Pd film deposited on a-Si. Crystalline regions are visible, corresponding either to fcc Pd or hex-Pd_2_Si, further supported by electron diffraction pattern (Figure 13b).

As first proposed in [105], the simultaneous occurrence of a tensile stress peak and resistivity drop at $h_{f}$ ~ 2 nm during growth of Pd on a-Si was ascribed to the crystallization of a Pd_2_Si interfacial compound. This scenario was confirmed and more precisely described in a recent work that combined simultaneously MOSS and XRD measurements using synchrotron facilities (beamline SIXS at SOLEIL) [145]. The authors could reveal that a nanocrystalline or amorphous Pd_2_Si layer is first formed, which suddenly crystallizes giving rise to the observed tensile peak and resistivity drop at 2.3 nm. Then, the Pd layer nucleates while the [111] textured Pd_2_Si layer continues to grow up to a thickness of 3.7 nm. The second drop in resistivity observed in Figure 12 around 3–4 nm could be the fingerprint of the formation of the elemental and more conductive Pd layer. As shown in Figure 12b, the tensile peak is better resolved and sharper at *T* = 355 K due to thermally-enhanced interfacial reaction. The similarities between the stress and resistivity curves recorded on a-Ge at *T* = 300 K and a-Si at *T* = 355 K point towards a higher reactivity between Pd and Ge atoms.

## 5. Conclusions and Outlook

The results presented herein demonstrate that non-invasive and easy to implement optical and electrical probes can be used as efficient in situ and real-time diagnostics to characterize the early growth stages of polycrystalline metallic films on semiconducting or insulating substrates. Examples are provided for a wide variety of metal/substrate systems for which different wetting behaviors and morphological evolutions are clearly revealed, primarily dictated by differences in surface mobility and interaction of the metal adatoms with the substrate layer. In the case of elemental metals deposited on weakly-interacting substrates (SiO_2_, a-C) and growing in 3D fashion (like Ag, Cu, or Pd), wafer curvature, spectroscopic ellipsometry, and electrical resistivity measurements are complementary in monitoring the relevant film formation stages, and determining morphological transition thicknesses corresponding to percolation and formation of a continuous layer. Through a systematic study of the dependence of these thicknesses with deposition rate *F* and substrate temperature *T*, the scaling behavior of these quantities has been established, from which two kinetic growth regimes could be identified. These regimes correspond to a coalescence-free and coalescence-controlled evolution of the island shapes.

More generally, the impact of deposition process parameters (working pressure, bias voltage, target-to-substrate distance) on film morphology can be directly evaluated by means of these real-time diagnostics, and establish the following empirical rules for controlling film morphological evolution: (i) for transparent conductors, it is necessary to form a percolated film at the lowest possible thickness, which can be achieved by decreasing substrate temperature, increasing deposition rate, or decreasing working pressure; (ii) opposite variations of the latter parameters decrease wetting and yield nanogranular films/supported nanoparticles. Application of a bias voltage may have an opposite effect on the onset of film percolation and formation of a continuous layer, depending on the type of sputtering discharge (DC vs. Hipims).

Our in situ and real-time nanoscale monitoring can be readily applied for studying the growth of other metals like Au, Pt, or Ti in the prospect of fabricating smart coatings capable to enhance the absorption of light in photovoltaics [146] or manage the light in novel dielectric–metal–dielectric transparent electrodes [147,148]. The developed methodology can also be extended to investigate the impact of alloying elements or minority species acting as surfactant on the film morphology and stress response. It also offers the possibility to explore more complex systems like transition metal nitrides, thin film metallic glasses or high-entropy alloys. Concurrently, it would be also very interesting to employ these real-time diagnostics for a better understanding of the mechanisms controlling solid-state dewetting of continuous layers [149,150,151], which is a promising route to fabricate nanogranular films.

The potential of coupling in situ and real-time stress and resistivity measurements is also leveraged to unveil structural transitions and interfacial reactions during growth of ultrathin transition metal (Fe, Mo, and Ta) and Pd layers on amorphous Si or Ge. These systems are characterized by a 2D growth morphology and the formation of an intermixed layer at the metal/Si (or Ge) interface over a thickness of ~1 nm. For Mo/Si and Fe/Si systems, the interfacial silicide compound is amorphous, and the metal layer crystallizes into its equilibrium bcc structure at a critical thickness of ~2 nm, as detected in real-time from the stress and resistivity transient features. Ta films grow instead in a tetragonal structure, with no visible change in the electrical and stress properties.

The overall results reviewed in the present article demonstrate that in situ, real-time optical and electrical techniques are powerful tools to monitor thin film growth at the nanoscale and gain insights on the correlation among atomic-scale mechanisms and resulting film morphology. From the view point of applications, these diagnostics could further contribute to optimize the growth of functional nanoscale layers and smart coating technologies relying on the integration of plasmonic devices and transparent electrodes on substrates beyond Si, such as glass, oxides, Van-der-Waals 2D crystals, or polymeric/flexible substrates [17,146,147,148,152,153,154]. To this end, their implementation may involve some technological improvement/modification of the actual set-up (for instance the curvature of a transparent substrate from laser-based deflectometry can be measured from the substrate backside coated with a reflective layer or by using other types of stress-sensors), as well as refinement of the optical modelling of ellipsometric data (to account for interband transitions in some metallic films, or optical anisotropy of some polymeric substrates). Combining these approaches with in situ X-ray-based techniques will enable us to get a detailed understanding of the structure formation and film morphology, which opens the path to engineering the film morphological and physical attributes by appropriate control of the process parameters.

## Figures and Tables

**Table 1:** Deposition conditions and characteristic morphological transition thicknesses for Ag, Cu and Pd films deposited by magnetron sputtering at *T* = 300 K on SiO_2_. The Ar working pressure is 0.3 Pa.

Element	Crystal Structure	Target Power (W)	Deposition Rate *F* (nm/s)	$T_{h}$	$h_{perc}$ (nm)	$h_{cont}$ (nm)
Ag	fcc	15	0.06	0.24	5.9 ± 0.1	12.4 ± 0.1
Cu	fcc	30	0.06	0.22	2.6 ± 0.1	8.2 ± 0.1
Pd	fcc	30	0.08	0.16	1.7 ± 0.1	5.9 ± 0.1

**Table 2:** Process parameters and physical quantities extracted from real-time stress and resistivity evolutions during growth of Fe, Mo, and Ta films at *T* = 300 K on a-Si. The Ar working pressure was fixed at 0.24 Pa.

Element	Crystal Structure	Target Power (W)	Deposition Rate *F* (nm/s)	$T_{h}$	Δf (J/m^2^)	ξ (nm)	$h_{perc}$ (nm)
Fe	bcc	60	0.06	0.16	2.3 ± 0.1	0.20 ± 0.06	0.30 ± 0.05
Mo	bcc	50	0.05	0.10	3.4 ± 0.1	0.30 ± 0.05	0.27± 0.05
Ta	β (A-15)	50	0.05	0.09	3.9 ± 0.1	0.39 ± 0.02	0.14± 0.05
